# Interaction between the Lentil Lipid Transfer Protein Lc-LTP2 and Its Novel Signal Ligand PI(4,5)P2

**DOI:** 10.3390/membranes10110357

**Published:** 2020-11-20

**Authors:** Daria Melnikova, Ivan Bogdanov, Tatiana Ovchinnikova, Ekaterina Finkina

**Affiliations:** 1 Science-Educational Center, M.M. Shemyakin and Yu.A. Ovchinnikov Institute of Bioorganic Chemistry, 117997 Moscow, Russia; d_n_m_@mail.ru (D.M.); contraton@mail.ru (I.B.); ovch@bk.ru (T.O.); 2 Department of Physicochemical Biology and Biotechnology, Moscow Institute of Physics and Technology (State University), 141701 Dolgoprudny, Russia; 3 Department of Bioorganic Chemistry, Lomonosov Moscow State University, 119234 Moscow, Russia

**Keywords:** lipid transfer protein, lentil, lipid binding, phosphatidylinositol (4,5)-bisphosphate, signaling, molecular docking

## Abstract

It is known that plant lipid transfer proteins (LTPs) bind a broad spectrum of ligands including fatty acids (FAs), phospho- and glycolipids, acyl-coenzyme A and secondary metabolites. In this work, we used protein−lipid overlay assays to identify new putative LTP ligands. In our experiments, the lentil lipid transfer protein Lc-LTP2 as well as LTPs from other plants were shown to bind phosphatidylinositol (4,5)-bisphosphate (PI(4,5)P2). Molecular modeling, 2-p-toluidinonaphthalene-6-sulphonate (TNS) displacement and liposome leakage experiments with Lc-LTP2 and its mutant analogs (R45A, Y80A, R45A/Y80A) were performed to investigate interactions between the protein and PI(4,5)P2. It was shown that PI(4,5)P2 initially interacted with the “bottom” entrance of the protein cavity and after complex formation the large polar head of this ligand was also oriented towards the same entrance. We also found that two highly conserved residues in plant LTPs, Arg45 and Tyr80, played an important role in protein-ligand interactions. Apparently, Arg45 is a key residue for interaction with PI(4,5)P2 during both initial contacting and holding in the protein cavity, while Tyr80 is probably a key amino acid playing an essential role in Lc-LTP2 docking to the membrane. Thus, we assumed that the ability of Lc-LTP2 to bind PI(4,5)P2 suggests the involvement of this protein in plant signal transduction.

## 1. Introduction

Lipids play an especially important role in plant cells. They not only function as barriers, but also take part in signal transduction, trafficking, morphological changes, and cell division via lipid-protein interactions. To date, many lipid-binding proteins have been identified in plants, including lipid transfer proteins (LTPs). LTPs are small, cationic proteins that are found in all land plants, encoded by large gene families, and expressed in most tissues [1,2]. Their abundance indicates a particular importance in plant life.

In LTPs, four conserved disulfide bridges stabilize compact spatial structures containing three or four α-helices and a central hydrophobic cavity suitable for binding varied hydrophobic ligands [3]. Most often, LTPs are not involved in high-affinity ligand interactions, as evidenced by the micromolar range of dissociation constants (Kd) of LTP-ligand complexes. In vitro experiments showed that a broad range of LTPs bound “standard” hydrophobic ligands such as phospho- and glycolipids, acyl-coenzyme A, fatty acids (FAs) (C12−C27), and prostaglandins [4]. The preferential ligands are, in most cases, 14−18 carbon FA chains. However, LTP’s in vivo ligand-binding repertoire is still rather under-investigated. To our knowledge, only the non-covalent complex of the peach Pru p 3 with a derivative of the alkaloid camptothecin bound to phytosphingosine [5], and the covalent complex of the barley LTP with oxylipin [6], have been isolated from natural sources. 

Plant LTPs are mainly synthesized as precursors of a signal peptide, and have extracellular localization. However, some of them can change their localization for an intracellular one, or function as GPI-anchored in membrane proteins [1]. LTPs are involved in a variety of plant processes due to their ability to bind and transfer different ligands. LTPs transport various hydrophobic molecules inside the cell, in the apoplast and intercellular space as well as through the phloem. These proteins take part in lipid metabolism, the formation of hydrophobic cover layers and protective secondary metabolite secretion. A number of data also indicate that LTPs apparently interact with signaling molecules, for example, with hormones such as jasmonic and gibberellic acids, and play an important role in signal transduction, intercellular interactions and activation of plant immune response [4]. However, much remains unclear. In this respect, identification of new possible LTP ligands as well as study of protein-ligand interactions may help provide molecular insight into the biological roles of these proteins in plants that are not clearly understood.

In a previous study we showed that Lc-LTP2 from the lentil *Lens culinaris* seeds binds and transfers a broad range of FAs (C12-C22) and lysolipids (PC and PG with different acyl chain lengths) [7]. We described the mechanism of lipid uptake from the LPPG (lyso-palmitoyl phosphatidylglycerol) micelle by Lc-LTP2, and demonstrated the roles of two residues belonging to the “bottom” entrance of Lc-LTP2, Tyr80 and Arg45, which can affect the microarchitecture of the protein hydrophobic cavity, ligand specificity and effectiveness of the FAs and lysolipid binding [8]. In this study, we identify a new putative Lc-LTP2 ligand and discuss the matter of the detected protein-lipid interactions.

## 2. Materials and Methods

### 2.1. Materials

1-palmitoyl-2-oleoyl-snglycero-3-phosphocholine (POPC) and 1-palmitoyl-2-oleoyl-sn-glycero-3-phosphoglycerol (POPG) were purchased from Avanti Polar Lipids (Alabaster, AL). 2-p-toluidinonaphthalene-6-sulphonate (TNS) and phosphatidylinositol (4,5)-bisphosphate (PI(4,5)P2) were purchased from Sigma-Aldrich (St. Louis, MO, USA). Membrane lipid strips (P-6002) came from Echelon Biosciences (Salt Lake City, UT, USA) The recombinant proteins were overexpressed in *Escherichia coli* and purified as described previously [8,9]. Homogeneity and the identity of the recombinant protein samples were confirmed by SDS-PAGE and MALDI mass spectrometry (Supporting Materials Figures S1 and S2). Rabbit polyclonal anti-Lc-LTP2-antibodies were obtained earlier [10].

### 2.2. Lipid Binding Assays

The protein-lipid overlay assays were performed using membrane strips with immobilized lipids (100 pM per spot). Detection of protein-ligand interactions was performed according to the manufacturer’s instruction. Briefly, the strips were treated sequentially with blocking buffer (2% nonfat dry milk in PBS-T), 5 mkg/mL LTP solution (in 0.2% nonfat dry milk in PBS), anti-Lc-LTP2 antibody solution (1:100 in 1% nonfat dry milk in PBS), anti-rabbit IgG horseradish peroxidase conjugated antibody solution (1:5000 in 1% nonfat dry milk in PBS) and chemiluminescent peroxidase substrate (Sigma). PBS-T (0.05% TWEEN-20) was used for membrane washing in each stage. Images were obtained by using the Gel Doc XR^+^ imaging system (Bio-Rad, Hercules, CA, USA). Recombinant proteins at different dilutions were spotted onto membranes as positive controls. As a negative control, the protein-lipid overlay assay without strip incubation with protein solution was performed. ELISA and immunoblotting for determination of antibody cross-reactivity was performed as described [10].

The ability of tested LTPs to bind PI(4,5)P2 was assayed using the fluorescent probe TNS. Lipid binding analysis was performed at 25 °C using an F-2710 spectrophotometer (Hitachi High Technologies America Inc., Pleasanton, CA, USA). The excitation and emission wavelengths were set at 320 and 437 nm, respectively. Before the initial fluorescence (F0) was recorded, 3 μM TNS with or without 10 μM of PI(4,5)P2 was incubated for 1 min in 2 mL of a measurement buffer (175 mM mannitol, 0.5 mM K_2_SO_4_, 0.5 mM CaCl_2_, and 5 mM MES at pH 7.0). The equilibrated fluorescence (F) was recorded after 2-min incubation with each of LTPs (final concentration of 4 μM). The results were expressed as a percentage of the LTP–TNS complex fluorescence calculated according to the formula [(F − F_0_)/F_C_] × 100%, where F_C_ is the fluorescence of the LTP–TNS complex in the absence of a lipid.

### 2.3. Bioinformatic Approaches to Study Interaction of PI(4,5)P2 with Lc-LTP2 Protein Variants

Two parent molecules were used for bioinformatic calculations: an NMR solution structure of apo-Lc-LTP2 (PDB ID: 2MAL) and an NMR solution structure of Lc-LTP2 complexed with 1-palmitoyl-2-hydroxy-sn-glycero-3-phosphoglycerol (LPPG) (PDB ID: 5LQV). Two sets of R45A, Y80A and R45A/Y80A mutant analogs of Lc-LTP2 were generated by means of the mutagenesis tool of the PyMOL 1.8.2.0 software (Schrödinger, LLC, New York, NY, USA) from both parent structures as templates [11]. 2D conformer of PI(4,5)P2 was obtained from the PubChem database (PubChem CID: 643962). Subsequent conversion of the 2D structure into three-dimensional space with energy minimization and geometry optimization was carried out using Open Babel v3.0.0 software [12]. Preparation of each protein variant for docking, including removal of water molecules, protonation, correction of errors and missing structure data, and assignment of partial charges using the AMBER ff14SB force field was carried out using the DockPrep tool of the UCSF Chimera v.1.4 software package (San Francisco, CA, USA) [13]. In order to use the protein structure obtained by the NMR solution (PDB: 5LQV) in docking experiments, the original LPPG ligand was removed from its complex with Lc-LTP2 during the receptor preparation steps. The docking box was chosen so that the whole protein molecule in the ribbon representation was entirely inside this box. Blind docking of PI(4,5)P2 based on the Lamarckian genetic algorithm (LGA) into each Lc-LTP2 variant was carried out using the AutoDock Vina tool of the UCSF Chimera v.1.4 software [14]. 2D ligand-protein interaction diagrams were generated with the Discovery Studio Visualizer v20.1.0.19295 software [15].

### 2.4. Calcein Release Assay

The calcein-entrapped small unilamellar vesicle (SUVs) composed of POPC; POPG; POPC:PI(4,5)P2 (at a 98:2 molar ratio), and POPG:PI(4,5)P2 (at a 98:2 molar ratio) were prepared by extrusion in the buffer containing 50 mM calcein, 10 mM HEPES, 200 mM NaCl, and 0.5 mM EDTA, with a pH of 7.5. Untrapped calcein was removed by gel filtration on a Sephadex G-50 column. The eluted calcein-entrapped vesicles were diluted to achieve the desired lipid concentration. Fluorescence was monitored with excitation at 490 nm and emission at 520 nm. Maximum fluorescence intensity (Ft) was determined by lysing vesicles in the presence of Triton X-100 (final concentration of 0.1%). The percentage of dye leakage caused by the protein was calculated as follows: dye leakage (%) = (F − F0)/(Ft − F0) × 100%, where F0 and F are the fluorescence intensity before and after the protein addition, respectively.

## 3. Results

### 3.1. Binding of Lipids

**Figure 1:**
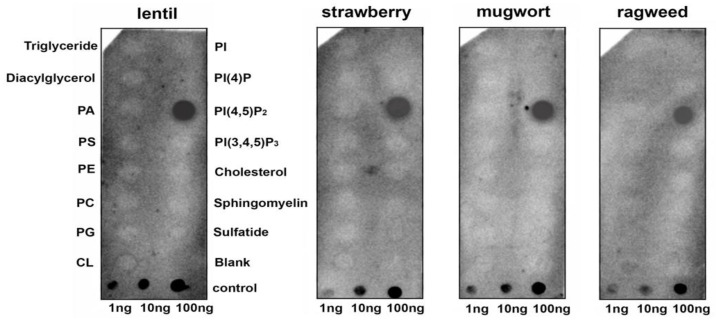
Lipid-binding assay of lipid transfer proteins (LTPs) from different plants using membrane lipid strips. PA, phosphatidic acid diC16; PS, phosphatidylserine diC16; PE, phosphatidylethanolamine diC16; PC, phosphatidylcholine diC16; PG, phosphatidylglycerol diC16; CL, 16:0 cardiolipin; Sulfatide, 3-sulfogalactosylceramide; PI, phosphatidylinositol diC16; PI(4)P, phosphatidylinositol (4)-phosphate diC16; PI(4,5)P2, phosphatidylinositol (4,5)-bisphosphate diC16; PI(3,4,5)P3, phosphatidylinositol (3,4,5)-trisphosphate diC16.

To assess the ability of a lentil Lc-LTP2 to bind different membrane lipids including those involved in signal transduction, a protein-lipid overlay assay was performed using membrane lipid strips (Echelon Bioscience). Detection of protein-lipid interactions was performed using polyclonal rabbit anti-Lc-LTP2 antibodies. Interestingly, it was found that Lc-LTP2 selectively bound to phosphatidylinositol (4,5)-bisphosphate (PI(4,5)P2) (Figure 1). Binding to other membrane lipids also containing two palmitoyl chains in their structure was not detected, except for a slight effect with the cardiolipin at an almost background level. To check whether LTPs from other plants bound this lipid, the recombinant proteins from strawberry (UniProt Q8VX12), mugwort (UniProt C4MGG9) and ragweed (UniProt O04004) were obtained. Cross-reaction of the polyclonal rabbit anti-LTP2 antibodies was confirmed by ELISA and immunoblotting methods (data not shown). Protein-lipid overlay assays with other LTPs from the abovementioned plants led to the same results (Figure 1).

**Figure 2:**
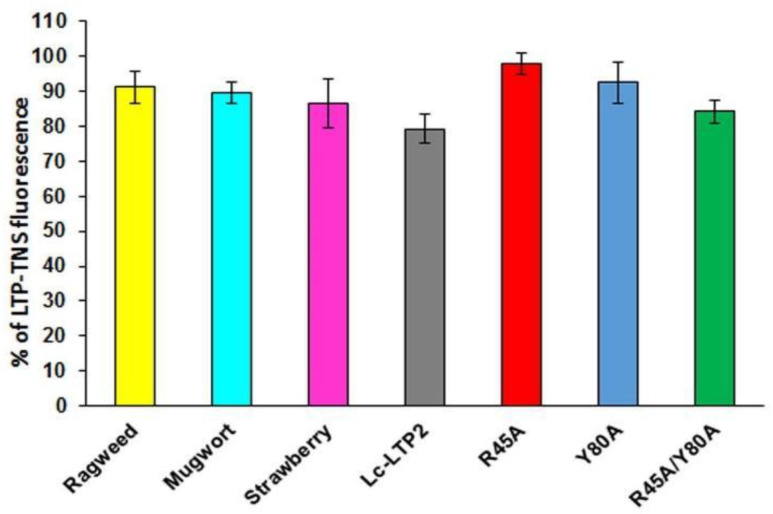
Effect of PI(4,5)P2 on the fluorescence level of the LTP-TNS complex. The results are expressed as the mean values (±SD) of the fluorescence percentage using the LTP–TNS complex without the lipid as a control.

To investigate whether LTPs bind to PI(4,5)P2 in aqueous solution, TNS displacement assays were performed (Figure 2). TNS fluorescent probe fluoresces intensely in hydrophobic environments but weakly in aqueous solution. Binding of TNS to the protein hydrophobic cavities resulted in an increase in fluorescence intensity, the value of which was taken as 100% for each protein. It was shown that PI(4,5)P2 was not easily accommodated in the cavities of LTPs from strawberry, mugwort and ragweed, reducing the fluorescence by 14%, 11% and 9%, respectively. PI(4,5)P2 more effectively displaced TNS from the Lc-LTP2 hydrophobic cavity (21%) compared to displacement from cavities of other tested LTPs. Previously obtained mutant analogs of Lc-LTP2 were also used in these experiments [8]. In the cases of the R45A/Y80A and Y80A analogs, the protein-TNS fluorescence was reduced by 16% and 8%, respectively. The reduction of the R45A-TNS fluorescence was not observed, suggesting the absence of PI(4,5)P2 binding with this mutant analog. 

### 3.2. Bioinformatic Approaches to Study of the Protein-Ligand Interactions

**Figure 3:**
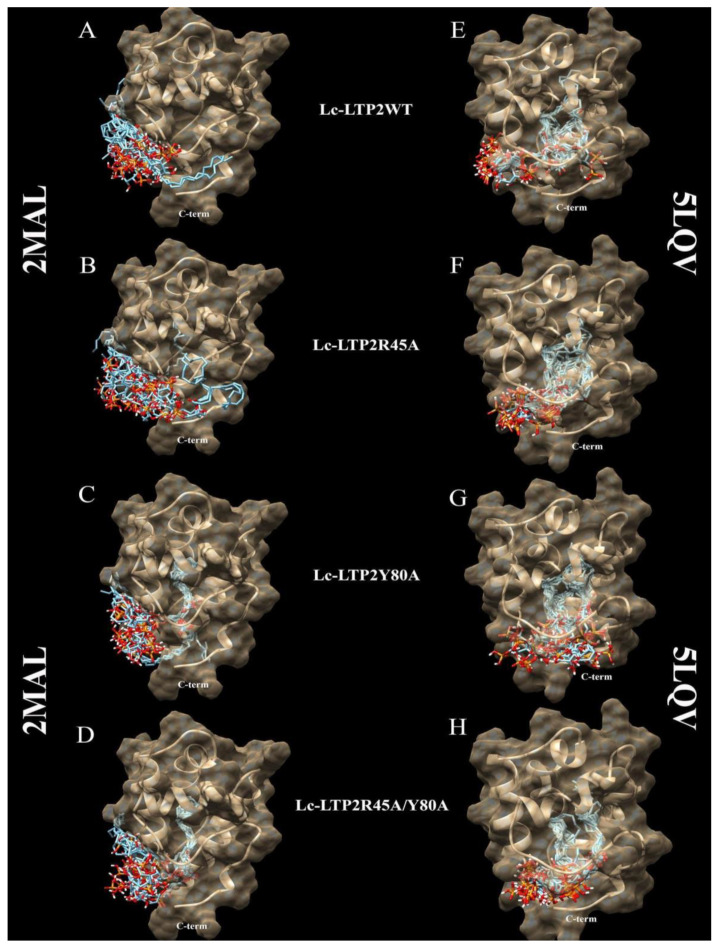
Spatial structures of wild-type Lc-LTP2 (**A**,**E**) and its mutant analogs R45A (**B**,**F**), Y80A (**C**,**G**), and R45A/Y80A (**D**,**H**) complexed with the PI(4,5)P2 conformations calculated by means of molecular docking. Visualization of the protein-ligand models was performed using the UCSF Chimera v.1.4 software.

**Figure 4:**
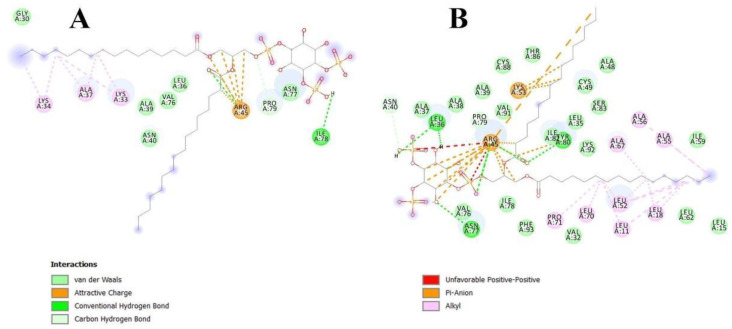
Interactions of apo-Lc-LTP2 (**A**) and liganded Lc-LTP2 (**B**) with PI(4,5)P2 based on computational molecular docking results.

It is known that plant LTPs do not have a ligand-binding site in the classical sense, as they can bind various ligands, accommodating them in the hydrophobic cavity. Blind molecular docking of a flexible ligand to a whole flexible receptor would be too complex and inaccurate if we used it to study LTPs interactions with double-chain lipids. Thus, in order to study the interaction of PI(4,5)P2 with the apo (PDB ID: 2MAL) and liganded (PDB ID: 5LQV) forms of the wild-type Lc-LTP2 and its R45A, Y80A and R45A/Y80A mutant analogs, blind molecular docking experiments were performed with the use of the PI(4,5)P2 (Figure 3 and Figure 4). In the case of apo-Lc-LTP2 (PDB ID:2MAL), the AutoDock Vina software found 10 conformations with affinity energy ranges between −4.0 and −3.5 kcal mol^−1^. AutoDock Vina placed all of them outside the hydrophobic cavity and close to the “bottom” entrance (Figure 3A). For the R45A mutant analog of apo-Lc-LTP2, AutoDock Vina found 10 conformations with affinity energy ranges between −4.1 and −3.6 kcal mol^−1^, two of which were also outside the cavity but with one acyl chain inside the pocket (Figure 3B). For the Y80A mutant analog of apo-Lc-LTP2, AutoDock Vina found 10 conformations with affinity energy ranges between −5.9 and −5.6 kcal mol^−1^, with one or two acyl chains that had entered the cavity from the “bottom” entrance with half-and-half representation (Figure 3C). For the double R45A/Y80A mutant analog of apo-Lc-LTP2, AutoDock Vina found 10 conformations of PI(4,5)P2 with affinity energy ranges between −6.2 and −5.5 kcal mol^−1^, all of them with one acyl chain inside the pocket and profound placement of the polar head at the “bottom” entrance (Figure 3D). As for the liganded (PDB ID:5LQV) structure, AutoDock Vina placed PI(4,5)P2 inside the hydrophobic pocket with affinity energy ranges between −6.6 and −6.2 kcal mol^−1^, with the polar head protruding out of the “bottom” entrance in all conformations. As for the R45A, Y80A, and R45A/Y80A mutant analogs of the liganded Lc-LTP2 (PDB ID:5LQV), AutoDock Vina placed the ligand similarly in the cavities of all the mutant analogs with affinity energy ranges between −7.3 and −6.4 kcal mol^−1^ (Figure 3E−H).

### 3.3. Effect of Lc-LTP2 and Its Mutant Analogs on SUVs

**Figure 5:**
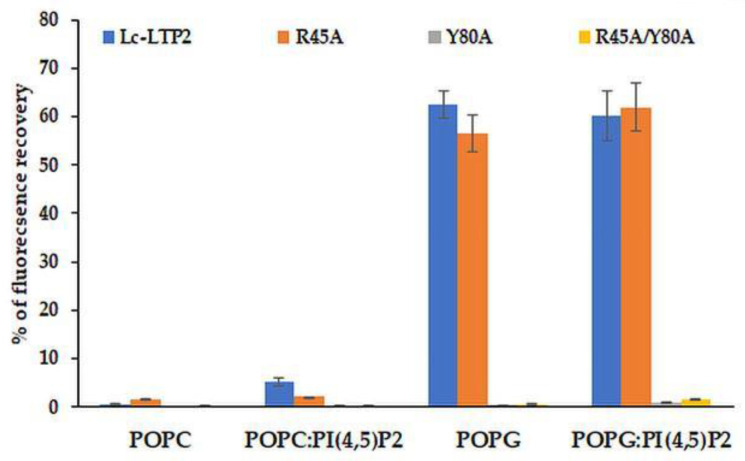
Percentage of calcein dye leakage from small unilamellar vesicle (SUVs) composed of POPC; POPG; and POPC:PI(4,5)P2 (at a 98:2 molar ratio); and POPG:PI(4,5)P2 (at a 98:2 molar ratio) upon addition of Lc-LTP2 and its mutant analogs. All proteins were used at a concentration of 20 μM. Each experiment was performed in triplicate. The results are expressed as the mean values (±SD) of the percentage of the fluorescence using the SUVs + Triton X-100 as a control.

Effects of Lc-LTP2 and its mutant analogs on the permeability of the POPG and POPC SUVs, as well as of those containing PI(4,5)P2, were investigated (Figure 5). In our previous work, we showed that Lc-LTP2 did not release calcein from POPC vesicles and induced a significant leakage of the POPG vesicles [7]. The R45A mutant analog was found to release calcein from such SUVs with an efficiency similar to Lc-LTP2, which indicated its interactions with the lipid bilayers. Interestingly, the R45A/Y80A and Y80A mutant analogs were not active at all in this assay. The presence of PI(4,5)P2 in SUVs slightly increased a release of calcein from the POPC:PI(4,5)P2 SUVs, but did not affect the POPG:PI(4,5)P2 SUVs’ permeability.

## 4. Discussion

The lipid composition of eukaryotic cells comprises sphingolipids, neutral lipids, glycolipids, and phospholipids with unique biophysical properties. Most of these lipids form the basis of biomembranes and take part in fat storage, but some of them are involved in signaling and membrane trafficking. Signal lipids are characterized by low abundance, rapid turnover, and transient increase of their synthesis in response to endogenous stimuli, which leads to the activation of different signaling pathways and triggering of subsequent cellular processes. Polyphosphoinositides (PPIs) and phosphatidic acid (PA) play an important role in plant signal transduction. Unlike animals, only five PPIs (i.e., PI(3)P, PI(4)P, PI(5)P, PI(3,5)P2, and PI(4,5)P2) are present in plants. These signaling lipids can specifically interact with proteins via selective lipid-binding domains that recognize the negatively charged lipid heads [16]. However, protein-ligand interactions depend not only upon electrostatic forces but also on spatial congruence. In the present work, we showed that PI(4,5)P2, mainly located in plasmatic membranes and regulating cellular functions through multiple mechanisms, is a novel signal ligand of the lipid transfer protein Lc-LTP2 from lentil seeds.

It is known that protein-lipid overlay assays are widely used for ligand identification and the study of lipid–protein interactions. As for plant LTPs, only one published paper has used this technique to demonstrate an ability of AtLtpI-4 from Arabidopsis to bind long-chain FAs [17]. In our experiments, we used hydrophobic nitrocellulose strips spotted with 15 different biologically significant membrane lipids including PA and PPIs. We showed that the lentil Lc-LTP2 as well as LTPs from other plants could bind PI(4,5)P2 (Figure 1). At the same time, no interaction with other membrane lipids also having two palmitoyl chains was found. We assumed that hydrophobic tails of the adsorbed lipid molecules are mainly anchored to the membrane, and hydrophilic heads were placed on the exposed side of hydrophobic membrane strips [18]. Primarily, surface electrostatic interactions of cationic plant LTPs were able to take place with the polar heads of lipids. Since we observed such a strong interaction with only PI(4,5)P2, we hypothesized that the ligand-protein conformational congruence was also a key for specific LTPs-lipid binding. 

Investigation of PI(4,5)P2 binding in aqueous solution demonstrated that Lc-LTP2 and LTPs from three other plants poorly displaced TNS from the protein cavities (Figure 2). We assumed that the cause lies in the conformational features of the formed protein-lipid complexes. On the one hand, incomplete space-filling of the protein hydrophobic cavities with the ligand might also take place. We previously observed such an effect for LMPG and LMPC, which had shorter acyl tails than LPPG and LPPC and did a worse job of displacing TNS [19]. On the other hand, the LTP-PI(4,5)P2 complexes in protein-overlay assays might be formed not only due to ligand interactions with the hydrophobic cavities, but also with other sites on the protein surfaces.

In order to clarify the situation, a computer modeling of the Lc-LTP2 complex was performed with PI(4,5)P2. In previous studies of NMR and molecular docking we showed that Lc-LTP2 formed complexes with anionic and zwitterionic phospholipids (DMPG, DHPC (1,2-diphytanoyl-sn-glycero-3-phosphocholine), LPPG and LPPC) in which the hydrophobic tails of the ligands were located inside the protein cavity, and their heads were located near the “top” entrance of the hydrophobic cavity [7]. In this work, we used the NMR structures of apo-Lc-LTP2 (PDB ID: 2MAL) for evaluation of an initial step of the lipid contact with the protein and the Lc-LTP2 spatial structure in its complex with LPPG (PDB ID: 5LQV]). This was used to model a possible final ligand location in the protein hydrophobic cavity (Figure 3). All the 10 calculated conformations showed that the ligand was located on the protein surface close to the “bottom” entrance of apo-Lc-LTP2 (Figure 3A). We found that Arg45 played a key role in the interaction with the ligand, forming a hydrogen bond and electrostatic interactions with the polar head of PI(4,5)P2 (Figure 4). We assumed that a similar ligand orientation in the complex with LTP might take place in the case of protein-overlay assays. As for the structure of liganded Lc-LTP2, hydrophobic tails of PI(4,5)P2 were located inside the protein cavity in all 10 calculated conformations, but its polar head was located outside the cavity close to the “bottom” entrance of the cavity. Thus, orientation of PI(4,5)P2 inside the Lc-LTP2 hydrophobic cavity near the “bottom” entrance was different compared with the abovementioned phospholipids, with one or two acyl chains accommodating at an opposite entrance. Based on these data, it can be concluded that Arg45 plays a significant role both in initiating surface interactions and holding the ligand in the hydrophobic cavity. Further, this might be applicable not only to Arg45, but also to Tyr80, Lys53 and some other amino acids that play an important role in protein-ligand complex stabilization (Figure 4).

Earlier, we showed that Lc-LTP2 induced a significant leakage of negatively charged POPG vesicles, but did not affect zwitterionic POPC vesicles or POPC:POPG vesicle permeability even at a ratio of 1:1 of these lipids. It was concluded that electrostatic interactions play a crucial role in the accumulation and destabilization of the protein vesicle surface [7]. Here, we included PI(4,5)P2 as a minor component (2%) of cell membranes in vesicle compositions. It was demonstrated that an inclusion of this lipid did not affect the permeabilizing activity of Lc-LTP2 in the case of POPG vesicles, but it had a slight effect on POPC vesicles (Figure 5). We assumed that the inclusion of PI(4,5)P2 does not affect the efficiency of POPG:PI(4,5)P2 (98:2) vesicle destruction by Lc-LTP2 because they had the negative charge across their surface, which probably makes the specific interaction of the protein with a polar head of PI(4,5)P2 impossible. Since POPC:PI(4,5)P2 (98:2) liposomes are almost uncharged, such a weak effect, in our opinion, could be associated not only with the electrostatic interaction of Lc-LTP2 and PI(4,5)P2, but mostly with the conformational congruence of this ligand to the protein.

In our previous work, we obtained three mutant analogs of Lc-LTP2: R45A, Y80A and R45A/Y80A, and showed an important role of Arg45 and Tyr80 residues located at the “bottom” entrance of the protein cavity in the binding of both FAs and lysolipids [8]. Here, we used these mutant analogs to study a role of Arg45 and Tyr80 residues in Lc-LTP2 interactions with PI(4,5)P2. We demonstrated that all the three mutant analogs bound this ligand worse than Lc-LTP2, especially R45A (Figure 2). These data confirm a significant role of Arg45 in PI(4,5)P2 binding as the residue that gave shape to the cavity architectonics. Tyr80 is probably also involved in the formation of a complex with the tested ligand, but to a lesser extent than Arg45. It is interesting to note that R45A/Y80A has the largest hydrophobic cavity among all the mutant analogs [8], and binds PI(4,5)P2 slightly better than other mutant analogs, probably due to a deeper placement in the protein cavity.

Molecular docking data showed that PI(4,5)P2 interacted with the “bottom” entrance of the wild-type protein and all of the mutant analogs of apo-Lc-LTP2. At the same time, an increase in the cavity volume (R45A < Y80A < R45A/Y80A) led to a deeper placement of the ligand in the cavity, which confirmed the results of the binding experiments (Figure 3). It is worth noting that PI(4,5)P2 orientation in the structures of all liganded mutant analogs was the same as that of Lc-LTP2. We assumed that PI(4,5)P2 has a very bulky polar head, which makes it impossible to place this ligand into the protein cavity in B and C orientations, as was demonstrated for LPPC, LPPG, DMPG and DHPC [7,8]. We previously described the mechanism of lipid uptake according to which Lc-LTP2 binds to the surface of LPPG micelle by its positively charged “bottom” entrance located near the C-terminal tail of the protein, after which the lipid penetrates into the protein cavity [7]. Based on our current data, we assume that in the case of signal function of PI(4,5)P2 such a mechanism of lipid uptake is less favorable upon contact with the membrane. 

In experiments with vesicles of different compositions we unexpectedly found that displacement of Arg45 did not affect the permeabilizing potential of Lc-LTP2 in the case of POPG. At the same time, the R45A/Y80A and Y80A mutant analogs had no effects on vesicle permeability at all. Thus, the absence of Arg45 presumably took part in an initial electrostatic interaction in which the vesicles could be compensated by the adjacently charged Lys81 and Lys53, which are also located close to the “bottom” entrance of Lc-LTP2. In contrast, the obtained data proved that the absence of Tyr80 cannot be compensated by any neighboring amino acids. At this point, we presume that Tyr80 is probably the key amino acid residue playing an essential role in Lc-LTP2-membrane docking.

## 5. Conclusions

In the present work, we showed that PI(4,5)P2 is a new possible ligand of lentil Lc-LTP2 and other plant LTPs using protein-lipid overlay assays. Results of PI(4,5)P2 binding in solution, molecular docking and liposome leakage experiments allowed us to assume that Lc-LTP2 binds this lipid in a different way than in cases of phosphatidylcholines and phosphatidylglycerols with one or two acyl tails. We showed that in the solution Lc-LTP2 apparently formed a complex with PI(4,5)P2 in which a polar head of the ligand is located outside the protein cavity close to the “bottom” entrance. At the same time, in the case of PI(4,5)P2 adsorbed on the membrane strip along with the electrostatic surface interaction of the protein with the lipid polar head, the conformational congruence of Lc-LTP2 with the ligan might be the most important factor behind the complex formation. We also demonstrated that Arg45, conserved in most plant LTPs, plays an important role in PI(4,5)P2-protein binding. Here, we suggested that Lc-LTP2 might bind to the polar head of PI(4,5)P2 without capturing the lipid from the cell membrane, after which a cascade of some signaling reactions could be triggered or other events might take place. Moreover, in addition to our previous data on the importance of another conservative residue, Tyr80, in the stabilization of the LTP-lipid complexes, we showed here for the first time a key role of this residue in LTP-membrane docking.

## Supplementary Materials

The following are available online at https://www.mdpi.com/2077-0375/10/11/357/s1. Figure S1: SDS-PAGE analysis of purified recombinant proteins was performed as described [10]. M—protein molecular weight marker; 1—Lc-LTP2; 2—R45A; 3—Y80A; 4—R45A/Y80A; 5—strawberry LTP; 6—ragweed LTP; 7—mugwort LTP, Figure S2: MALDI mass spectra of purified recombinant proteins obtained in a linear mode using Ultraflex III MALDI-TOF mass spectrometer (Bruker) equipped with a UV Nd:YAG laser (355 nm) and LIFT MS/MS unit. The measured m/z values correspond to the calculated masses of protonated molecular ions [M + H]^+^ of the proteins stabilized by four disulfide bonds.

## Abbreviations

DHPC	1,2-dihexanoyl-snglycero-3-phosphocholine
DMPG	1,2-dimyristoyl-sn-glycero-3-phosphoglycerol
FA	fatty acid
LPPC	lyso-palmitoyl phosphatidylcholine
LPPG	lyso-palmitoyl phosphatidylglycerol
LTP	lipid transfer protein
PI(4,5)P2	phosphatidylinositol (4,5)-bisphosphate
POPC	1-palmitoyl-2-oleoyl-snglycero-3-phosphocholine
POPG	1-palmitoyl-2-oleoyl-sn-glycero-3-phosphoglycerol
TNS	2-p-toluidinonaphthalene-6-sulphonate
SUV	small unilamellar vesicle
