# Supplementary material for: Interaction between the Lentil Lipid Transfer Protein Lc-LTP2 and Its Novel Signal Ligand PI(4,5)P2
Source: Membranes (Basel). 2020 Nov 20;10(11):357. doi: 10.3390/membranes10110357 (PMC7699592; doi:10.3390/membranes10110357)
Supplement: Supplementary file 1 [file membranes-10-00357-s001.pdf]

Supplementary Materials:

Interaction between the Lentil Lipid Transfer Protein Lc-LTP2 and Its Novel Signal Ligand PI(4,5)P2

Daria Melnikova [1,2], Ivan Bogdanov [1], Tatiana Ovchinnikova [1,2,3] and Ekaterina Finkina [1,*]

[1] Science-Educational Center, M.M. Shemyakin and Yu.A. Ovchinnikov Institute of Bioorganic Chemistry, 117997 Moscow, Russia; d_n_m_@mail.ru (D.M.); contraton@mail.ru (I.B.); ovch@bk.ru (T.O.)

[2] Department of Physicochemical Biology and Biotechnology, Moscow Institute of Physics and Technology (State University), 141701 Dolgoprudny, Russia

[3] Department of Bioorganic Chemistry, Lomonosov Moscow State University, 119234 Moscow, Russia

*Correspondence: finkina@mail.ru; Tel.: +7-495-335-42-00

Supplementary Figures

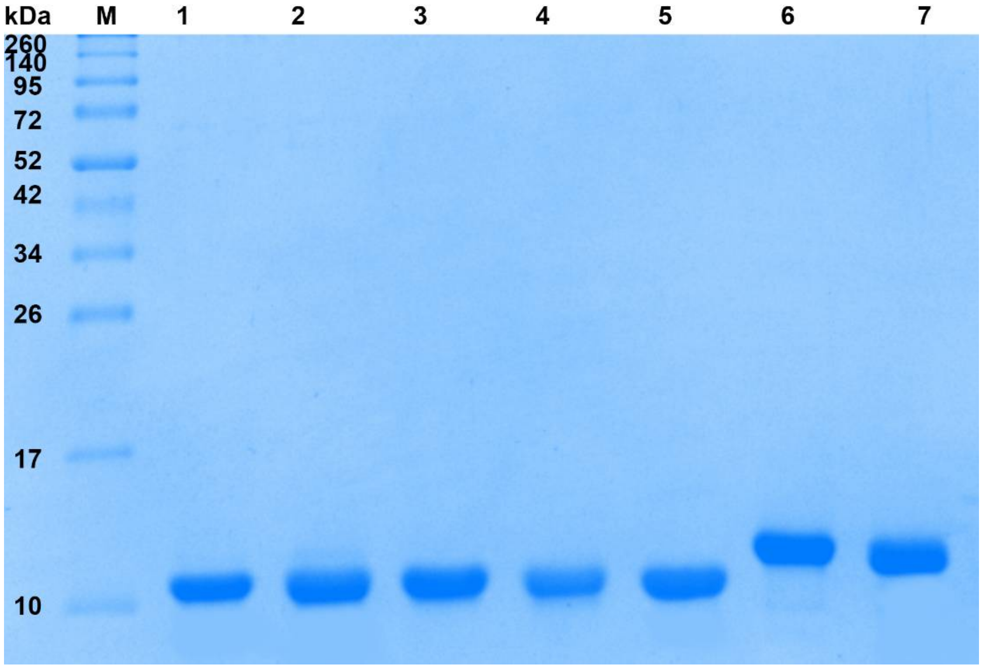

Figure S1. SDS-PAGE analysis of purified recombinant proteins was performed as described [10]. M - protein molecular weight marker; 1 - Lc-LTP2; 2 - R45A; 3 - Y80A; 4 - R45A/Y80A; 5 – strawberry LTP; 6 – ragweed LTP; 7 – mugwort LTP.

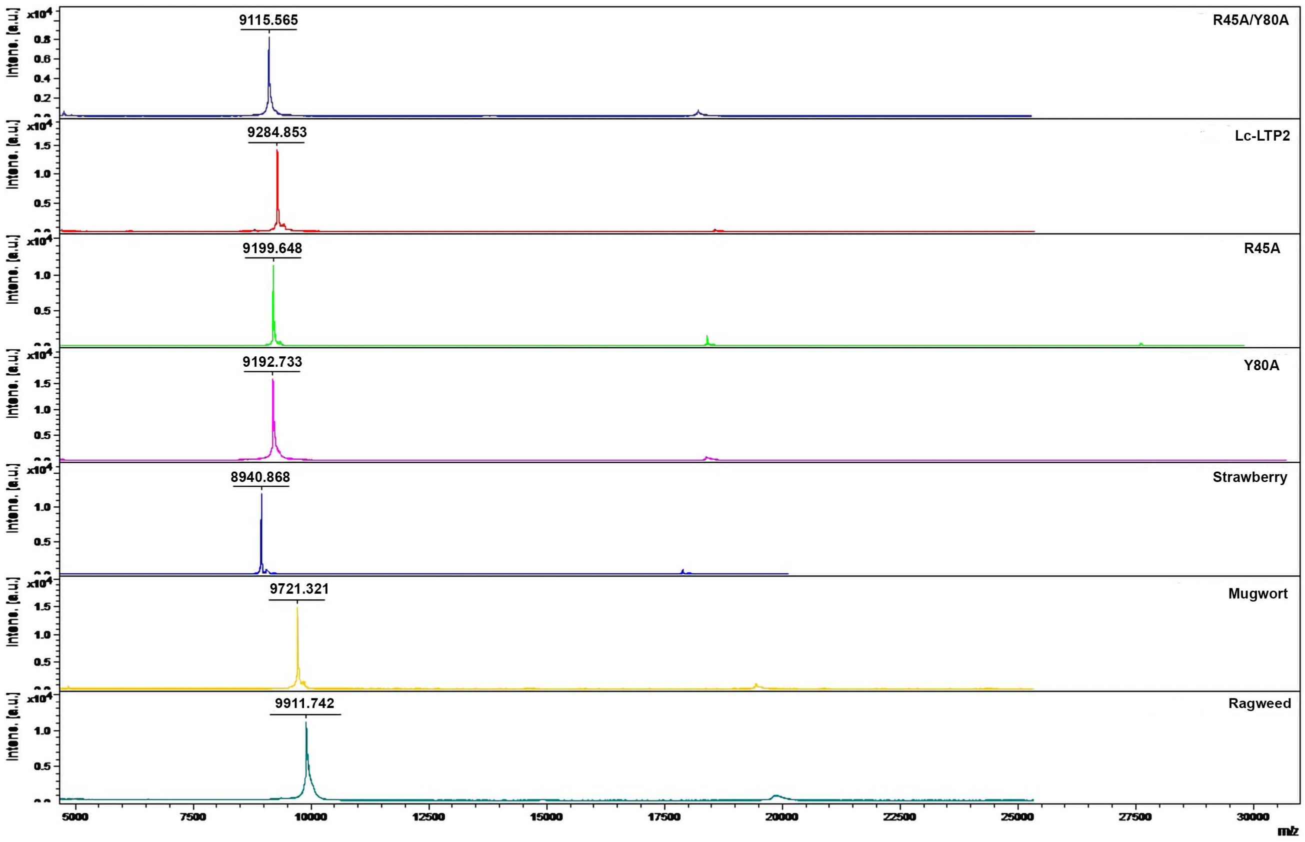

Figure S2. MALDI mass spectra of purified recombinant proteins obtained in a linear mode using Ultraflex III MALDI-TOF mass spectrometer (Bruker) equipped with a UV Nd:YAG laser (355 nm) and LIFT MS/MS unit. The measured m/z values correspond to the calculated masses of protonated molecular ions $[M+H]^+$ of the proteins stabilized by four disulfide bonds.
